# Human T cell lymphotropic virus type 1 (HTLV-1) infection increases the CD4 count in human immunodeficiency virus type 1 (HIV-1) infected patients submitted to antiretroviral therapy (ARV)

**DOI:** 10.1186/1742-4690-11-S1-P62

**Published:** 2014-01-07

**Authors:** Filipe FA Rego, Luiz CJ Alcantara, Tulio de Oliveira

**Affiliations:** 1 CPqGM/FIOCRUZ, Salvador, Bahia, Brazil; 2 University of KwaZulu-Natal, Durban, KwaZulu-Natal, South Africa

## 

To understand the impact of HTLV-1 in the CD4 counts in HIV coinfected patients from KwaZulu-Natal, 382 HIV-1 plasma samples with ARV failure were screened for HTLV-1. Out of 382 samples, eight were reactive in the EIA and seven were confirmed as HTLV-1 positive by PCR. The data from those patients was obtained in the RegaDB and was analyzed regarding the CD4 count using the STATA software. The prevalence of coinfection was 1.8% (7/382). The baseline CD4 count did not show statistical difference between groups before treatment. The overall CD4 count median during the ARV in coinfected patients (301cells/uL) was higher than in HIV-1 infected patients (232cells/uL *p*=0.0002). The CD4 count did not behave different between the groups along the time. This observation might be due to the higher treatment time in coinfected patients (median 1764days vs. 1459days, p<0.0001), provided that in the end of therapy they were failing on the ARV but the paired analyze with viral load must be done whereas there is an inverse correlation between CD4 count and viral load. There was no difference regarding the age and gender between the groups. HIV-1/HTLV-1 co-infection seems to change the CD4 count ratio between positive and negative individuals on ARV.

